# Dynamics of working memory process revealed by independent component analysis in an fMRI study

**DOI:** 10.1038/s41598-023-29869-2

**Published:** 2023-02-18

**Authors:** Magdalena Fafrowicz, Anna Ceglarek, Justyna Olszewska, Anna Sobczak, Bartosz Bohaterewicz, Monika Ostrogorska, Patricia Reuter-Lorenz, Koryna Lewandowska, Barbara Sikora-Wachowicz, Halszka Oginska, Magdalena Hubalewska-Mazgaj, Tadeusz Marek

**Affiliations:** 1 Department of Cognitive Neuroscience and Neuroergonomics, Institute of Applied Psychology, Jagiellonian University, Lojasiewicza Street 4, 30-348 Krakow, Poland; 2 Department of Psychology, University of Wisconsin-Oshkosh, Oshkosh, WI USA; 3 Department of Psychology of Individual Differences, Psychological Diagnosis and Psychometrics, Faculty of Psychology, SWPS University of Social Sciences and Humanities, Warsaw, Poland; 4 Chair of Radiology, Medical College, Jagiellonian University, Krakow, Poland; 5 Department of Psychology, University of Michigan, Ann Arbor, MI USA; 6 Department of Drug Addiction Pharmacology, Maj Institute of Pharmacology, Polish Academy of Sciences, Krakow, Poland

**Keywords:** Neuroscience, Cognitive neuroscience, Learning and memory

## Abstract

Human memory is prone to errors in many everyday activities but also when cultivating hobbies such as traveling and/or learning a new language. For instance, while visiting foreign countries, people erroneously recall foreign language words that are meaningless to them. Our research simulated such errors in a modified Deese-Roediger-McDermott paradigm for short-term memory with phonologically related stimuli aimed at uncovering behavioral and neuronal indices of false memory formation with regard to time-of-day, a variable known to influence memory. Fifty-eight participants were tested in a magnetic resonance (MR) scanner twice. The results of an Independent Component Analysis revealed encoding-related activity of the medial visual network preceding correct recognition of positive probes and correct rejection of lure probes. The engagement of this network preceding false alarms was not observed. We also explored if diurnal rhythmicity influences working memory processes. Diurnal differences were seen in the default mode network and the medial visual network with lower deactivation in the evening hours. The GLM results showed greater activation of the right lingual gyrus, part of the visual cortex and the left cerebellum in the evening. The study offers new insight into the mechanisms associated with false memories, suggesting that deficient engagement of the medial visual network during the memorization phase of a task results in short-term memory distortions. The results shed new light on the dynamics of working memory processes by taking into account the effect of time-of-day on memory performance.

## Introduction

In new language learners, errors that include the incorrect recall of foreign words are common. For example, in the Esperanto language, it is easy to confuse the words antidotoj (antidotes) and anekdotoj (anecdotes). The short-term memory task we use here resembles situations in a foreign country where we try to recall words that are foreign and thus relatively meaningless. These “pseudo” words are useful and important, for example, street or station names during sightseeing, but we fail by uttering phonetically similar words. We were interested in identifying neuronal activity associated with the performance of memory distortions that resemble the aforementioned mistakes and examining potential of time-of-day effects on these mechanisms. We use a modified version of the Deese-Roediger-McDermott (DRM) paradigm^1,2^ and time-of-day approach to investigate this problem.

The concept of working memory has figured prominently in the study of cognition over the last 50 years. Working memory is responsible for the temporary retention and management of information required for a range of higher cognitive abilities such as problem-solving, comprehension, and reasoning^3–5^. Overall, working memory is thought to be important for goal-directed behaviors, which require maintenance, selection, and sequencing functions^6,7^. The terms “working memory” and “short-term memory” are becoming synonymous to some extent^8^ with working memory seen as a "multi-component system that holds and manipulates information in short-term memory"^9^. Such a view is adopted in this work. The Multicomponent Working Memory Model proposed by Baddeley and Hitch (1974)^10^ is one of the most influential early models and has been widely used to describe working memory processes^11–14^. According to this model, the central executive coordinates information stored in various independent “slave” subsystems, most notably, the visuospatial sketchpad and the phonological loop. The former is responsible for retaining and processing information in a visual and spatial form, and is typically used for navigation and visuospatial reasoning. The phonological loop consists of two distinct parts—the phonological store and the articulatory control process—that actively maintain spoken and written verbal material. Evidence suggests this subsystem also contributes to acquiring new words during the process of foreign language learning through the engagement of phonological short-term memory^15,16^. A conventional way to evaluate the above subsystem memory is the pseudoword repetition test, consisting of words which do not exist in a given language, however are phonologically similar to the ones that do.

Many experimental paradigms have been used to study human memory and its failings. One of the most popular is Deese-Roediger-McDermott (DRM) paradigm (for the review, see: REF^17^), used for studying memory distortions, because of its simplicity, controllability, and flexibility^18^. In this paradigm, participants study a list of related words, each of which converges on a non-studied critical lure. When tested, participants often recall and/or recognize the critical lure as having been presented, resulting in a form of memory distortion^1,2,19–22^. In DRM adjusted to study short-term memory (ST-DRM), the delay between encoding and recall/recognition lasts for several seconds^23,24^. This variant of the DRM task allows to effectively separate the encoding (presentation of the memory set) and retrieval processes (remembering whether or not the recognition probe was presented in the preceding memory set). While most DRM tasks focus on the distorting effects of semantic similarity, memory distortions due to phonological similarity (based on surface/structural content) are also investigated^17,18^. In a review by Chang and Brainerd (2021)^18^, the authors showed that there are differences between semantic and phonological distortions examined using DRM paradigm under both short-term and long-term memory conditions as well as due to the test modality (auditory versus visually presented items). McBride et al. (2019)^25^ demonstrated that false memories are stronger for phonological than for semantic lists in short-term memory. Regarding the stimulus modality, Olszewska et al. (2015)^26^ showed that phonological illusions were lower when the list was presented visually.

While numerous neurocognitive models of working memory have been proposed^27^, the recent dynamic-processing model of working memory endeavors to account for a differing sets of brain-based findings that have been difficult to reconcile with any single model^28^. The dynamic-processing model proposes that the relevant information is retained based on dynamically-coded representations and maintenance processes that vary according to the task demands (e.g. interference), task rules, and behavioral goals. Likewise, the associated neural substrates vary dynamically according to the representational and processing demands of the tasks and associated behavioral goals^29^.

Various brain imaging studies have established the neural bases of human working memory and documented the critical involvement of the fronto-parietal network in these process^14,30,31^. Logie et al. (2003)^32^ showed brain activation in the inferior parietal gyrus as well as the left middle frontal gyrus while processing phonological material in working memory. In the work utilizing laminar functional MRI (lfMRI), Sharoh and collegues (2019)^33^ revealed unique connectivity patterns during visually presented both words and pseudowords reading. The difference was seen in the depth-dependent BOLD signals of the left occipitotemporal sulcus in a way that to words responded deep parts of this structure and to pseudowords—superficial and middle parts. The majority of studies in the magnetic resonance (MR) environment aimed to investigate the distortions of long-term memory (for the reviews, see: REF^34,35^). Several regions such as the medial superior frontal gyrus, left precentral gyrus as well as left inferior parietal cortex were related with false memory retrieval, regardless of the type of material. Regarding short-term false memory, activity of the dorsolateral prefrontal cortex was revealed during correct rejection of related lures in semantic material while true recognition was associated with the activation in the left fusiform gyrus^26^ as well as in figural material demonstrating hippocampal areas activations related to fewer correct rejections of lures^36^ and higher false recognition-related activity of prefrontal and visual cortices in young adults^37^. Many neuroimaging studies investigated the formation of true and false memories focusing on the sensory reactivation theory^38^, however recent evidence strongly transforming this concept, showing that memory representations differ from perceptual representations and pointing out memory retrieval as constructive not reproductive act^39^.

Previous resting-state fMRI studies revealed that brain functional architecture also changes dynamically according to the time-of-day^40^. Accordingly, the other aim of the present study is to determine if there are diurnal changes in the dynamic of working memory processes. Many studies revealed the time-of-day effects on cognition, in which the optimal time for the performance of a certain task was evaluated (for the reviews, see: Ref^41,42^). Three experimental procedures—constant routine, forced desynchrony, and time-of-day protocols—are essentially used for the investigation of daily fluctuations of cognitive processes^41,43^. When constant routine and forced desynchrony procedures are performed in the strictly controlled, laboratory environment, in the time-of-day paradigm participants (examined at optimal or/and nonoptimal time-of-day) between experimental sessions carry out their routine activity. One of the experimental designs here is the chronotype-based paradigm, which allows investigating over the normal working day the temporal fluctuations of performance related to individual’s circadian preference (chronotype). In addition, we considered the genetic factors, which have been shown to contribute both diurnal preference and homeostatic regulation of sleep. There is some evidence that polymorphism of *PER3* gene is associated with individual differences in circadian and sleep phenotypes. The *PER3* 4-repeat allele has been linked with “eveningess”, whereas *PER3* 5-repeat allele with “morningness” and greater homeostatic sleep pressure (e.g.^44–46^). More information regarding the chronotypes-based paradigm used in our research is presented in the Supplementary Information. Although several studies indicated a diurnal variation in the performance of working memory tasks^36,47–49^, the results of these studies are not always consistent and it is still an underexplored area of research. From the neuroimaging perspective, the studies revealed differences in brain activation according to the time-of-day. Schmidt et al. (2015)^48^ showed higher thalamic activity in the morning and decreased activity in the ventrolateral prefrontal cortex and premotor areas from the morning to the evening hours using the n-back paradigm. Singh et al. (2022)^50^ demonstrated higher brain activations in the morning session during working memory task performance.

The literature reviewed showed that most fMRI results related to both short-term and long-term false memory were obtained with use of the general linear model (GLM). Yet, exploratory approaches, such as the Independent Component Analysis (ICA), may shed light on the underexplored aspects of diurnal cognitive variability. ICA is a data-driven approach, which aims for decomposition of a multivariate signal into additive subcomponents^51^. The method is considered a special case of blind source separation that resembles the cocktail party effect where one sound is isolated from the noise^52^. To the best of our knowledge, neither brain imaging nor an exploratory analytic approach like ICA has been used to investigate time-of-day effects on short-term memory processes.

Taken together, the present study was designed to investigate the neural mechanisms underlying the generation of distortions in working memory and to evaluate if the time-of-day influences these mechanisms using the DRM paradigm, together with a time-of-day protocol, and the ICA approach to the fMRI data. We expect that the obtained results will offer new insights into the dynamics of working memory processes. These results may also be interesting in the context of learning new languages to the extent such processes are similar to the memorization of a random group of syllables.

## Materials and methods

### Participants

Online advertisement on the Jagiellonian University website and Facebook was the primary recruitment method for the study. A total of the 5354 young, healthy volunteers participated in the first stage of selection and completed assessment of diurnal preference as measured by the Chronotype Questionnaire^53^, night sleep quality as measured by the Pittsburgh Sleep Quality Index (PSQI^54^), and daytime sleepiness as measured by the Epworth Sleepiness Scale (ESS^55^). Four hundred fifty-one participants identified as morning or evening chronotypes reporting no excessive daytime sleepiness (ESS score ≤ 10 points) and good sleep quality (PSQI score ≤ 5 points) went through the next stage of selection. The selection criteria for chronotypes were: above 15 points in amplitude scale from Chronotype Questionnaire and between 8 and 19 for morning types and between 26 and 32 for evening types in morningness/eveningness scale from Chronotype Questionnaire.

In the next stage of selection all participants underwent genotyping to identify *PER3* VNTR polymorphisms affecting circadian typology and sleep homeostatic drive (for the review, see: Ref^46^). To perform genotyping, mouth swabs were collected from all of the participants. More information about this procedure is presented in the Supplementary Information. Only individuals who were homozygous for the *PER3* 4- alleles (evening chronotypes) and *PER3* 5-alleles (morning chronotypes) were included in the study. Further exclusion criteria included dyslexia (four participants eliminated), drug, caffeine, alcohol or nicotine dependence (three participants excluded), shift work (one person excluded), and having been on a flight passing more than two time zones within the past 2 months (one participant eliminated). The total sample for the fMRI study consisted of 66 young, healthy native Polish speakers who reported no dyslexic symptoms and fulfilled the selection criteria: age between 20 and 35 years, right-handedness according to the Edinburgh Handedness Inventory (EHI^56^), normal or corrected-to-normal vision, regular time-of-day schedule without sleep debt, no neurological or psychiatric disorders, and no MRI contraindications. Informed, written consent was provided by all participants prior to completion of the study procedures. The sleep–wake schedule of participants was monitored using the MotionWatch 8 actigraphy during the week preceding the study as well as the days of brain imaging. Imaging data from eight participants were excluded, three due to the excessive movements, defined as exceeding a 4° rotation or 4 mm translation on any axis and five due to the lack of errors in at least one condition of the task. Hence, 58 participants (37 females; mean age = 24.4, SD = 3.68) divided into two groups: 29 morning types (MT) and 29 evening types (ET) were included in the analyses (see above for the selection criteria). The participants were remunerated for participation in the experiment. The study was conducted in accordance with the Declaration of Helsinki and approved by the Research Ethics Committee at the Institute of Applied Psychology at the Jagiellonian University. Demographics, questionnaires and actigraphy results are provided in Table 1.

**Table 1:** Demographics, questionnaires and actigraphy results.

Variables	MT (*N* = 29)		ET (*N* = 29)		*Sign*
	*M*	*SD*	*M*	*SD*	
Sex (M/F)	10/19		11/18		-
Age (years)	24.52	4.04	24.24	3.37	ns
ME	15.62	2.64	28.35	2.07	**
AM	21.88	3.60	22.48	3.02	ns
ESS	5.34	2.33	7.70	3.40	*
PSQI	3.14	0.99	3.31	1.20	ns
EHI	86.13	13.87	90.73	14.03	ns
Declared waketime (hh:mm)	6:58 AM	64 min	7:37 AM	51 min	*
Declared bedtime (hh:mm)	11:21 PM	54 min	00:06 AM	48 min	*
Declared length of perfect sleep (hh:mm)	08:50	61 min	08:25	41 min	ns
Actigraphy-derived waketime (hh:mm)	7:36 AM	74 min	8:35 AM	72 min	*
Actigraphy-derived bedtime (hh:mm)	11:55 PM	60 min	1:01 AM	56 min	*
Actigraphy-derived length of real sleep (hh:mm)	06:33	44 min	06:28	31 min	ns
*PER3* polymorphism	5/5		4/4		-

Mann–Whitney U test, *p < 0.05, **p < 0.001, *ns* not significant.

*MT* morning types, *ET* evening types, *ME* morningness/eveningness scale from Chronotype Questionnaire, *AM* amplitude scale from Chronotype Questionnaire, *ESS* Epworth Sleepiness Scale, *PSQI* Pittsburgh Sleep Quality Index, *EHI *Edinburgh Handedness Inventory, *M* mean, *SD* standard deviation.

### Stimuli

The experimental task was based on the modified short-term memory DRM paradigm^23,24^ using pseudowords as memoranda, with a distractor task filling the brief retention interval.

Memory items: 550 pseudowords—pronounceable meaningless letter strings—containing Polish syllables were used to create memory sets characterized by phonological (and visual) similarity. All stimuli in one set were made up of similar Polish morphemes and started or ended with the same syllable. The number of characters in the stimuli varied from 5 to 9 for two syllables pseudowords (mean length 6.11), from 5 to 10 for three syllables pseudowords (mean length 7.68), and from 7 to 11 for four syllables pseudowords (mean length 8.65).

**Figure 1:**
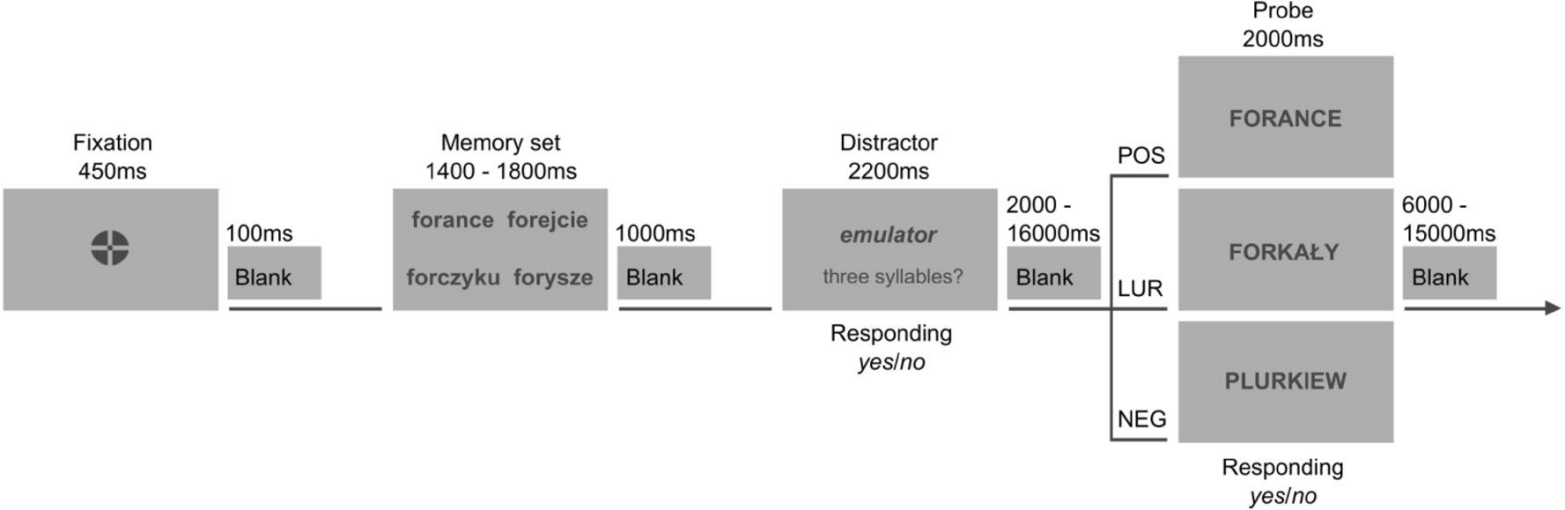
The sequence of stimuli in the task with an example set of pseudowords and distractor. *POS *positive probe, *LUR *lure probe, *NEG *negative probe.

Distractor items: 120 concrete Polish adjectives and nouns served as distractors occurring between memory sets and probes (see Fig. 1). The number of characters in two-syllable words varied from 5 to 9 (mean length 6.5), in the three-syllable words from 5 to 12 (mean length 8.02), and in the four-syllable words from 8 to 11 (mean length 9.75).

All stimuli (memory sets, distractors and memory probes) were presented in Calibri 22-point font via a mirror on an MR-compatible LCD screen (NordicNeuroLab, Bergen, Norway) with a refresh rate of 60 Hz and a resolution of 800 × 600 pixels. Each item in the memory set appeared at one of four possible locations at 11° and 18° of visual angle in x-axis and 7° and 9° in y-axis, whereas the distractor and memory probe were presented at the center of the screen. The colors used were dark gray (RGB 72, 72, 72) in the foreground and light gray (RGB 176, 176, 176) in the background.

### Task

Two equivalent versions (version A and version B) of the experimental task were used, each consisting of 60 trials and differing only in terms of stimuli sets and distracting words. In total there were 120 memory sets consisting of four pseudowords (two, three, or four syllables) each and 120 distractors consisting of two, three, or four syllable words. As distractors we used low frequency words in Polish language based on the dictionary by Kazojc (2011)^57^.

Each participant performed the task twice—during morning (MS) and evening (ES) sessions in a 3 T Siemens Magnetom Skyra MRI scanner. Counterbalancing took place allowing all four possible pairing between the two task versions (A and B) and the two sessions (MS and ES) across participants, in a manner that half of participants performed version A in the morning and version B in the evening, whereas the opposite was true for the other half of participants. The possibility of the practice effect was rejected, no differences were found in both the error ratio and the reaction times between the first session held for each participant (MS or ES) and the second. Each experimental trial began with a fixation point for 450 ms and was followed by a blank screen presented for 100 ms. A set of four pseudowords was presented simultaneously for 1400, 1600 or 1800 ms (encoding phase), depending on the number of syllables (two, three or four syllables, respectively). 1000 ms followed offset of the memory set, then a distractor item (word) appeared at the center of the screen for 2200 ms. An interval of 2000–16,000 ms (mean duration 6097 ms) followed the distractor task, and then a single stimulus/pseudoword served as the memory probe, which appeared for 2000 ms (retrieval phase). The memory probe was of three possible types: positive probe (a studied item from the immediately preceding list), lure (a pseudoword phonologically similar to the items studied in the immediately preceding list) and a negative probe (a new pseudoword, unrelated to those previously studied in the immediately preceding list). For each set, the lure was selected on the basis of a pilot study (a stimulus that was the closest one to the average of a whole set) and the negative probe-based on specific criteria (number of syllables and stimuli beginnings/ends). Mean duration of the inter-trial interval was 8403 ms (range: 6000–15,000 ms). The task consisted of 60 sets of stimuli followed by 25 positive probes, 25 lures, and ten negative probes. The number of sets in each version of task was as follows: two and four syllables—15 sets each; three syllables—30 sets. The number of distractors in each task: two- and four-syllables words—6 each; three-syllables words—48. The experimental task lasted 22 min. Figure 1 shows the sequence of events in the task.

Participants were instructed to memorize each set of four stimuli in order to recognize the presented pseudoword a few seconds later. During the distractor task, participants were asked to indicate whether or not a word consisted of three syllables. In response to the memory probe participants indicated whether they remember that the item appeared in the directly preceding set. For both the distractor task and the memory task, participants responded by pressing a button in their right hand for ‘yes’ responses and a button in their left hand for ‘no’ responses.

Within each version of the experiment, there were six lists created in a manner such that each stimulus could be followed by each probe type (e.g. in each list, out of 60 sets presented to a participant, ten different probes were followed by a negative probe). The lists were counterbalanced across participants. Within each list the order of stimuli was randomized but the order of probe types (positive, lure or negative), inter-stimulus intervals and inter-trial interval was fixed and the same for each participant (using the Optseq, FS-Fast analysis tools by D. Greve, Charlestown, MA). During the task, a single memory set could appear only once. The tasks were prepared using E-Prime 2.0 (Psychology Software Tools, Pittsburgh, PA, USA).

### Procedure

Participants completed the study in three visits: a training session, a morning session, and an evening one. During the training session—started at 5 PM one day before the first experimental session—the participants were extensively trained on the experimental procedure and types of stimuli to avoid the influence of a learning process on their performance. The training session consisted of three parts. In the first each participant was informed about the course of the experiment. Next sample trials were used to familiarize the participant with the experimental task procedure and trial types. Six experimental trials (2 positive probes, 2 lure probes, and 2 negative ones) were presented to the participant. There was no time limit to familiarize with each trial component—participant pressed a key to proceed to the next part of the trial. The subject could familiarize with trial components as many times as he/she needed. In the third part of the training session a whole-task training approach was used. The participants responded to both the probe and distractor by pressing a key with the right hand (response ‘yes’) or left hand (response ‘no'). Stimuli for training differed to those used for the experimental tasks. The tasks were presented on a HP Compaq laptop with a 15.4-in LCD monitor, with the screen resolution set at 1280 × 768 pixels and a screen refresh rate of 60 Hz. The presentation and data recording were controlled with E-prime 2.0 software (Psychology Software Tools, Pittsburgh, PA, USA). A research assistant who accompanied the participants during the training clarified any questions about the task. The participant could complete the task as many times as he/she needed. No feedback for correct or incorrect response was given, but the research assistant who assisted the participant during the training session encouraged him/her to practice the task until they felt well-trained and comfortable with the task. Participants abstained from alcohol (48 h) and caffeine (24 h) before each fMRI session and on the experimental days. During experimental days participants could engage in non-strenuous activities. The night before the morning session, they slept in rooms located in the building of the MR laboratory. The tasks were performed by morning-type participants between 09:49 AM and 10:11 AM (*SD:* 1 h 08 min) in the morning and between 18:44 PM and 19:06 PM (*SD:* 1 h 12 min) in the evening. Evening-type participants performed the tasks between 11:09 AM and 11:31 AM (*SD:* 1 h 24 min) in the morning and between 20:17 PM and 20:39 PM (*SD:* 1 h 25 min) in the evening.

### fMRI data acquisition

MRI data was acquired using a 3 T Siemens Skyra MR System with a 64-channel coil. For anatomical reference, a T1-weighted MPRAGE sequence was performed (TR = 2.3 s, TE = 2.98 ms, FA = 9°, 176 sagittal slices, slice thickness = 1.1 mm, FOV = 256 × 256 mm). For the BOLD imaging, a T2*—weighted EPI sequence was used (TR = 1.8 s, TE = 27 ms, FA = 75°, 34 slices with interleaved acquisition, voxel size = 4 × 4 × 4 mm, slice thickness = 4 mm, inter-slice gap = 0 mm, FOV = 256 × 256 mm). The 736 volumes were acquired during task performance. Participants’ eye movements were monitored using an eye tracking system (Eyelink 1000, SR research, Mississauga, ON, Canada).

### Behavioral data analysis

Statistical analyses were performed using Statistica 6.0 software (StatSoft, Inc., 2001) and R software version 4.0.0 (R Core Team, 2017). Behavioral analyses focused on hits (*yes* responses to positive probes) and false alarms (*yes* responses to critical lures and *yes* responses to negative probes) as well as on reaction times (RTs) to: hits, misses, correctly rejected critical lures and negative probes, and false alarms for critical lures and negative probes.

We performed signal detection analyses using dʹ as an estimate of sensitivity^58^. Following other studies^59–62^ two measures of sensitivity were computed using the individual participants’ data: (1) estimates of sensitivity comparing hits (*yes* responses to studied probes) to false alarms (*yes* responses to critical lures), which reflects item-specific memory and (2) estimates of sensitivity where false alarms were treated as hits (*yes* responses to critical lures) and were compared to unrelated probes treated as false alarms (*yes* responses to unrelated probes), which reflects gist-based memory. Each of these indices of sensitivity was treated as dependent variables in separate analyses of variance (ANOVAs), with chronotype (MT vs. ET) as a between-subjects factor and time-of-day (morning vs. evening) as a within-subjects factor.

### fMRI data analysis

#### Preprocessing

Data preprocessing was performed using the Statistical Parametric Mapping software package (SPM12, Welcome Department of Imaging Neuroscience, UCL, London, UK; www.fil.ion.ucl.ac.uk/spm/) and DPABI (V4.2) implemented on MATLAB (Mathworks, Inc., MA, USA). Scans were slice-timed corrected and realigned by inclusion of field maps. Following motion correction, each individual’s structural T1-weighted image was co-registered and spatially normalized to Montreal Neurological Institute (MNI) space, as well as resampled at 3 mm^3^ using B-Spline Interpolation. The normalized volumes were smoothed using 6-mm FWHM Gaussian kernel to increase the signal-to-noise ratio of the data. Then the signal from white matter and cerebrospinal fluid was regressed out and the band-pass filtering (0.01–0.08 Hz) was applied.

#### ICA

Preprocessed images were analyzed with the Group ICA of fMRI Toolbox (GIFT) software package (v4.0b; Medical Image Analysis Lab, University of New Mexico; http://icatb.sourceforge.net/groupica.html). Spatial ICA was performed for two sessions and the spatial maps, as well as associated time courses, were produced separately for morning and evening sessions. The optimal number of components was estimated using the Minimum Description Length (MDL) criteria in the GIFT software package. A two-step principal component analysis (PCA) was executed to lower the imaging data dimensionality. Independent component estimation was performed using the Infomax algorithm and repeated 50 times in ICASSO (http://research.ics.tkk.fi/ica/icasso) to assess the consistency of the components. For each independent component (IC), the ”centroid” (i.e. the most stable result) was determined following the agglomerative hierarchical clustering with average-linkage criterion and its consistency was calculated with a cluster quality index (Iq) ranging from 0 to 1. Subject-specific spatial maps and associated time courses were back-reconstructed using the GICA3 method provided in the GIFT software package. To identify non-artifactual ICs, the visual inspection of IC spatial patterns as well as frequency inspection of time courses was performed, based on the prior^63–65^. Then the time courses of thirteen selected intrinsic connectivity networks (ICNs) were regressed with the design matrix (see GLM section) created with regressors from the task (onsets of probes—hits, misses, false alarms, correct rejection of lure and negative probes for encoding and retrieval phases) to establish which networks were engaged in the task. The regression step was done using ‘mvregress’ function in Matlab environment. Next, the beta values were averaged for every ICN across the subjects and modelled separately for encoding and retrieval phases. Positive beta values meant the activation of a given network, while negative ones—its deactivation. The one-sample *t*-test on mean beta values for MS and ES was performed to see which networks were engaged in the task with false discovery rate (FDR) correction for multiple comparisons at *p* < 0.05 level using the Benjamini–Hochberg procedure. The paired *t*-tests for MS and ES were conducted for every ICN to define the differences in activation/deactivation of ICNs in distinct time-of-day with FDR correction for multiple comparisons at *p* < 0.05 level using the Benjamini–Hochberg procedure.

#### GLM

Additionally, the preprocessed data were analyzed using the General Linear Model (GLM) implemented on SPM12. In the first level of statistical analyses, the conditions were defined by their stimulus onsets, for each subject and session the design matrix was created with regressors from the task (hits, correct rejection of lure and negative probes, erroneous responses for positive (misses) and lure (false alarms) probes for encoding and retrieval phase and “others” regressor which contain individual erroneous responses for negative probe and events without the response and the realignment parameters as the regressors of no interest. The regressors were modeled by a boxcar function (locked to the onset of the memory set in the encoding phase and memory probe in the retrieval phase with duration of 2000 ms) convolved with a hemodynamic response function and contrasted against each other, which resulted in eight contrasts for each subject and session (hits > false alarms, false alarms > hits, correct rejection of lures > false alarms and false alarms > correct rejection of lures at encoding and retrieval). The negative probe was treated as a control condition due to the small number used in the task (10) and was not included in contrasts. In the second level of analysis (group-level), the paired t-tests using the above-mentioned contrasts for MS vs. ES were conducted to ascertain the differences in activations of brain structures in distinct time-of-day. The results are presented at cluster-wise *p* < 0.05 level with FDR correction for multiple comparisons and a cluster size of at least 20 voxels. Anatomical labeling of significantly activated clusters was performed using WFU Pickatlas software, extension to SPM12 (Functional MRI laboratory—Wake Forest University School of Medicine, Winston Salem).

### Ethics statement

The study was conducted in accordance with the Declaration of Helsinki and approved by the Research Ethics Committee at the Institute of Applied Psychology at the Jagiellonian University. The participants signed the written informed consent before the data gathering.

## Results

### Behavioral results

The linear mixed model was conducted with six response types (hits; misses; correct rejection and false alarms for lure probes; correct rejection and false alarms for negative probes) as fixed effects and participant as random effects to test for any differences in RTs between conditions of the task. The model was constructed using the lme function of the nlme R package (version 4.0.0) with restricted maximum likelihood. The probes without the response were not included in the analysis. An ANOVA was performed on the model. The significant effect of condition F(1,5) = 113.46, p < 0.0001 was revealed. The model indicated the significant differences between all response types (p < 0.001). The post hoc HSD Tukey test indicated the differences between correct rejection of negative and misses (p = 0.002) and between correct rejection of negative and false alarms for lure (p = 0.01). Table 2 showed mean RTs for all response types.

**Table 2:** Mean reactions times for six response types.

Response type	N	M	SD
Hits	2086	1256	395
Misses	797	1493	465
Correct rejection of lure	1990	1372	415
False alarms for lure	891	1439	413
Correct rejection of negative	1098	1145	343
False alarms for negative	53	1219	244
All	6915	1322	418

*M* mean, *SD* standard deviation, *N* number of observations.

A two by two ANOVA with time of day (morning vs. evening) as a within-subjects factor and chronotype (MT vs. ET) as a between-subjects factor was conducted on measures of sensitivity (d') for item-specific memory and gist-based memory. Table 3 presents measures of sensitivity (d') calculated for morning and evening time-of-day as well as morning and evening types.

**Table 3:** Measures of sensitivity (d′) of phonologically similar pseudowords for morning- and evening-types at different time-of-day (morning and evening) calculated for item-specific memory and memory for gist.

	MT		ET	
	MS	ES	MS	ES
	*M* (*SD*)	*M* (*SD*)	*M* (*SD*)	*M* (*SD*)
Item-specific memory				
Positive probe—critical lure	1.35 (0.59)	1.15 (0.65)	1.22 (0.66)	1.20 (0.61)
Gist-based memory				
Critical lure—negative probe	0.80 (0.79)	0.88 (0.70)	1.09 (0.60)	1.37 (0.58)

*MT *morning type, *ET *evening type, *MS *morning session, *ES *evening session, *M *mean, *SD *standard deviation.

#### Item-specific memory

There was no main effect of chronotype F(1, 56) = 0.05, p = 0.82, no main effect of time-of-day F(1, 56) = 1.78, p = 0.19 and no interaction F(1, 56) = 1.024, p = 0.32 in sensitivity associated with discriminating positive probes from critical lures.

#### Gist-based memory

There was a main effect of chronotype F(1, 56) = 8.25, p = 0.006, η^2^ = 0.13 showing greater reliance on gist among evening types (M = 1.23, SD = 0.61) than morning ones (M = 0.84, SD = 0.74). Neither a main effect of time of day F(1, 56) = 2.62, p = 0.11 nor an interaction F(1, 56) = 0.78, p = 0.38 were significant.

Next, we tested RT differences between times of day at which the study was performed and between chronotypes for each probe type: correctly recognized positive probe, correctly rejected lure probe and correctly rejected negative probe. For each probe type we performed 2 (chronotype: MT vs. ET) × 2 (time-of-day: morning vs. evening) ANOVA on RT and for all three probe types we revealed no main effects and no interactions (all Fs < 2, ps > 0.1).

### fMRI results

#### ICA results

Thirteen ICNs were extracted from the data: the anterior (medial prefrontal cortex, anterior cingulate gyrus) and posterior DMN (angular gyrus, posterior cingulate gyrus, precuneus), the left and right fronto-parietal network (fronto-parietal cortices), auditory network (superior temporal gyrus, Heschl’s gyrus), language network (inferior frontal gyrus, superior temporal gyrus), the medial (fusiform gyrus) and lateral (superior and middle occipital gyrus) visual network, occipital pole (inferior occipital gyrus), insular network, sensory-motor network (precentral gyrus, supplementary motor area), executive network (medial frontal gyrus, anterior cingulate gyrus) and dorsal attention network (intraparietal sulcus). The MNI coordinates of structures belonging to the given network are presented in Supplementary Information (Table S1).

**Figure 2:**
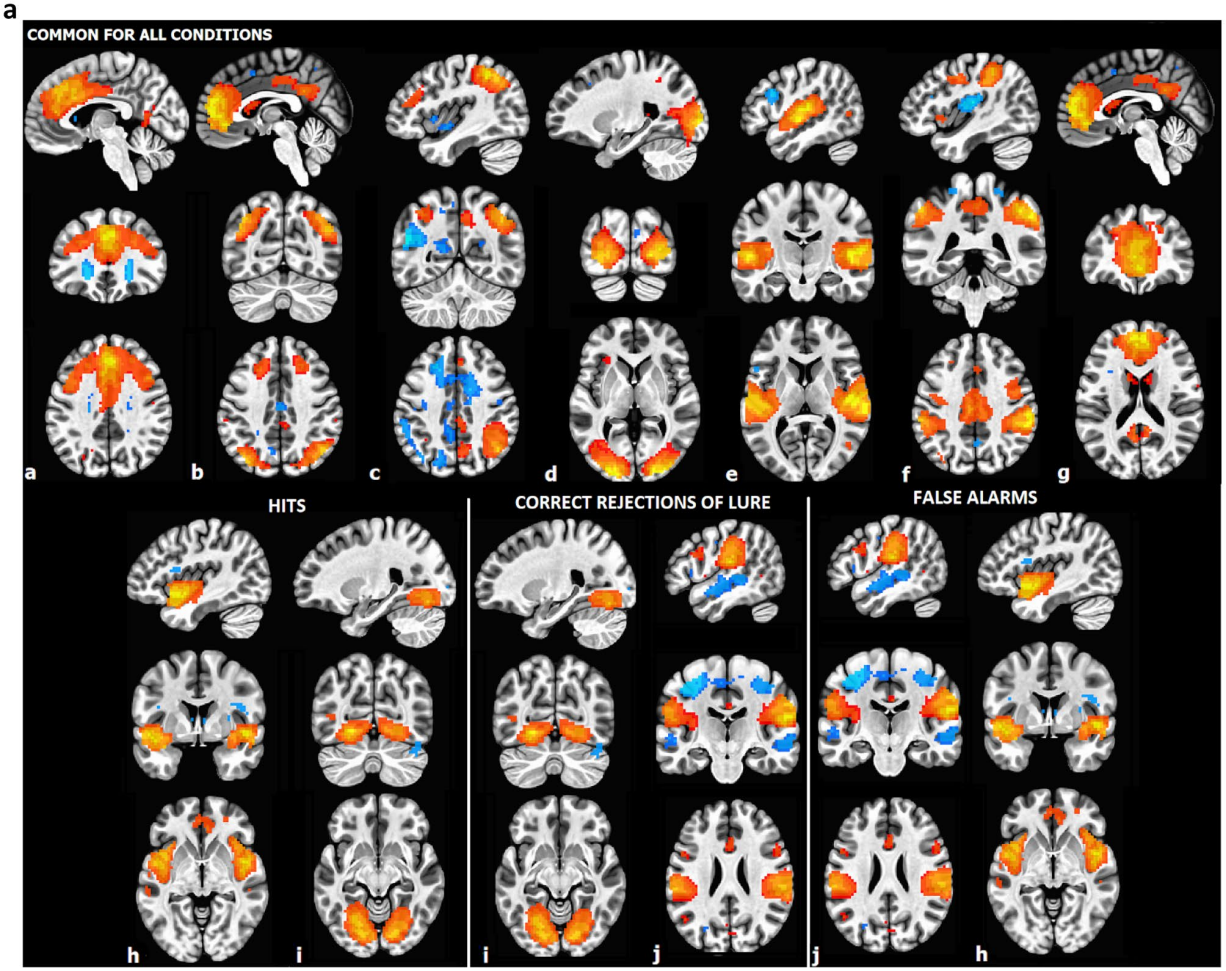

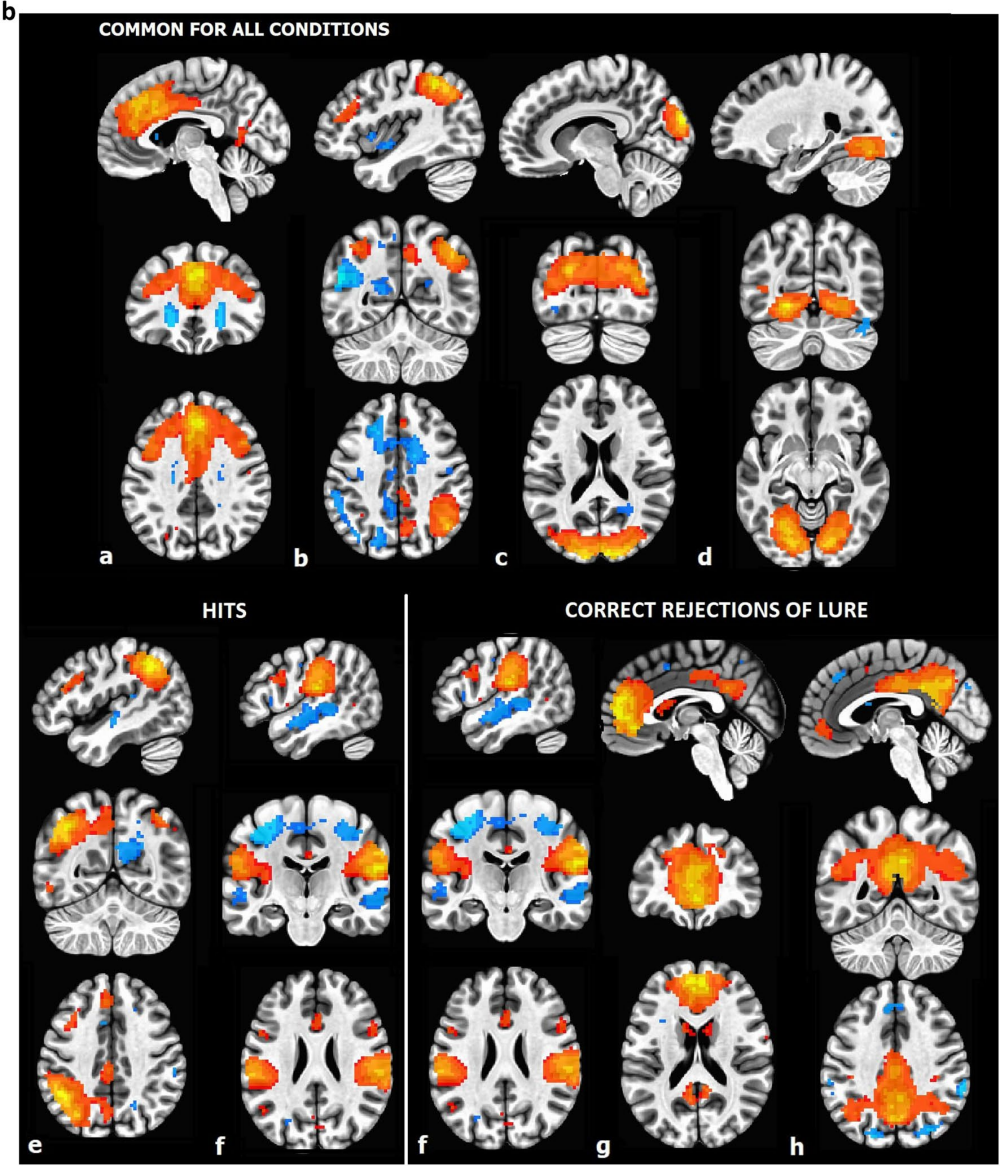
(**a**) The brain networks engaged in the encoding phase of the phonological task, common for all conditions and distinct for each of them (FDR corrected at *p* < 0.05 level). a—Executive network, b—dorsal attention network, c—right fronto-parietal network, d—lateral visual network, e—auditory network, f—sensory-motor network, g—anterior default mode network, h—insular network, i—medial visual, j—language network. (**b**) The brain networks engaged in the retrieval phase of the phonological task, common for all conditions and specific for hits and correct rejection of lure probes (FDR corrected at *p* < .05 level). a—Executive network, b—right fronto-parietal network, c—lateral visual network, d—medial visual network, e—left fronto-parietal network, f—language network, g—anterior default mode network, h—posterior default mode network.

In the encoding phase, for all conditions (hits, correct rejection of lures and false alarms) the activation of executive, right fronto-parietal and sensory-motor networks and the deactivation of anterior default mode, auditory and dorsal attention networks was evident. Furthermore, the analysis revealed activation of the medial visual network and deactivation of the insular network for hits, deactivation of the language network and activation of the medial visual network—for correct rejection of lure, and deactivation of insular and language network—for false alarms (Fig. 2a). For other conditions, no differences were found.

**Figure 3:**
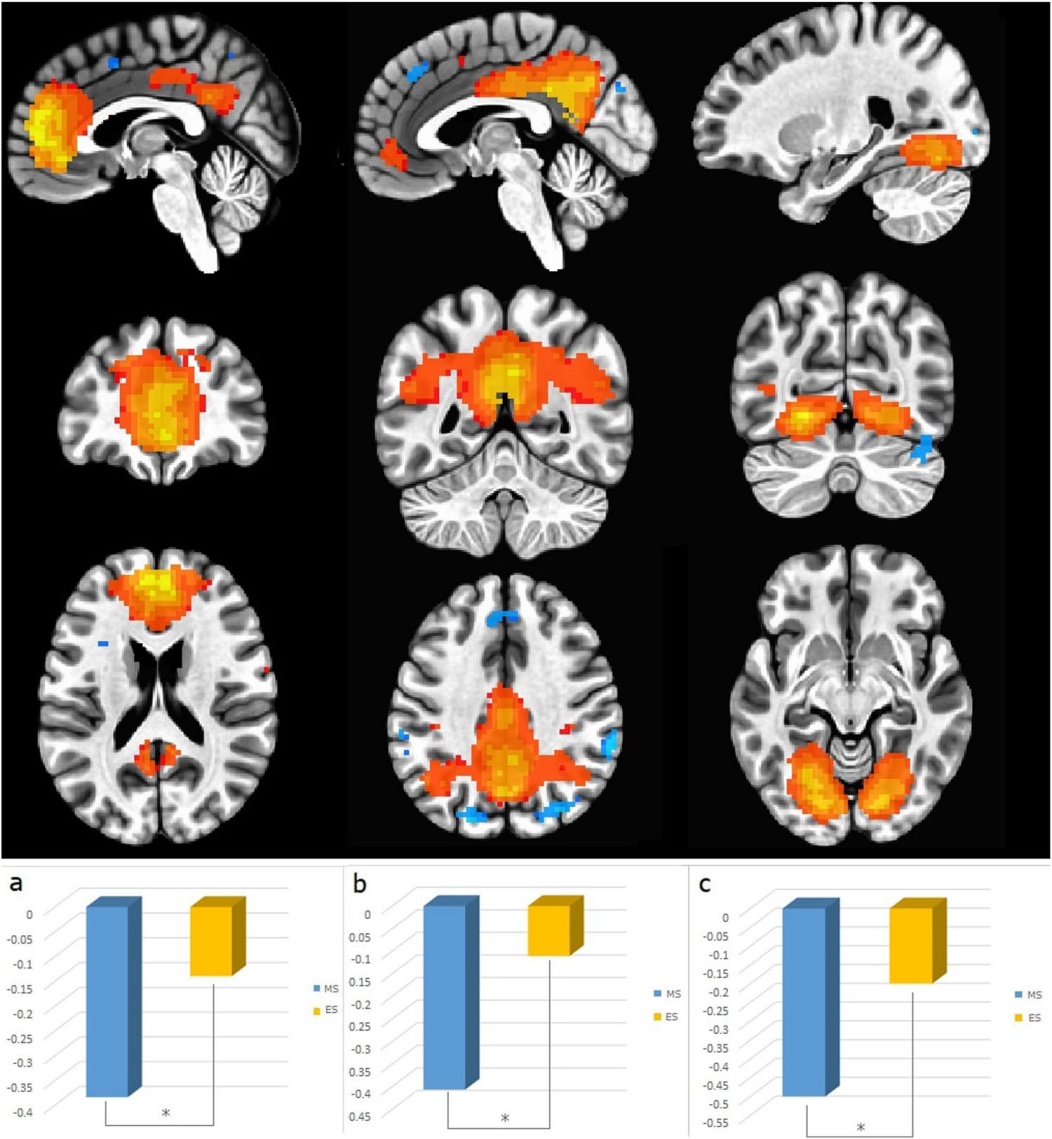
ICNs with diurnal differences during correct rejection of lure in the retrieval phase. Each network showed higher deactivation in the morning session compared to the evening session. The paired t-tests with FDR correction at *p* < 0.05 level were performed. (**a**) Anterior default mode network, (**b**) posterior default mode network, (**c**) medial visual network.

In the retrieval phase, for all conditions the activation of executive and right fronto-parietal networks and deactivation of lateral and medial visual networks was evident. Furthermore, for hits the activation of the left fronto-parietal networks was evident, and for correct rejection of lures—the deactivation of anterior and posterior default mode networks and activation of language network (Fig. 2b). It is worth emphasizing that no distinguishing networks were detected for false alarms during the retrieval phase. No differences were evident for engagement of ICNs between morning- and evening-type participants. The diurnal differences (MS vs. ES) were evident only for correct rejection of lure in the retrieval phase for the anterior and posterior default mode networks and the medial visual network—all exhibited more deactivations in the morning (Fig. 3). For other conditions, no differences were found.

#### GLM results

**Figure 4:**
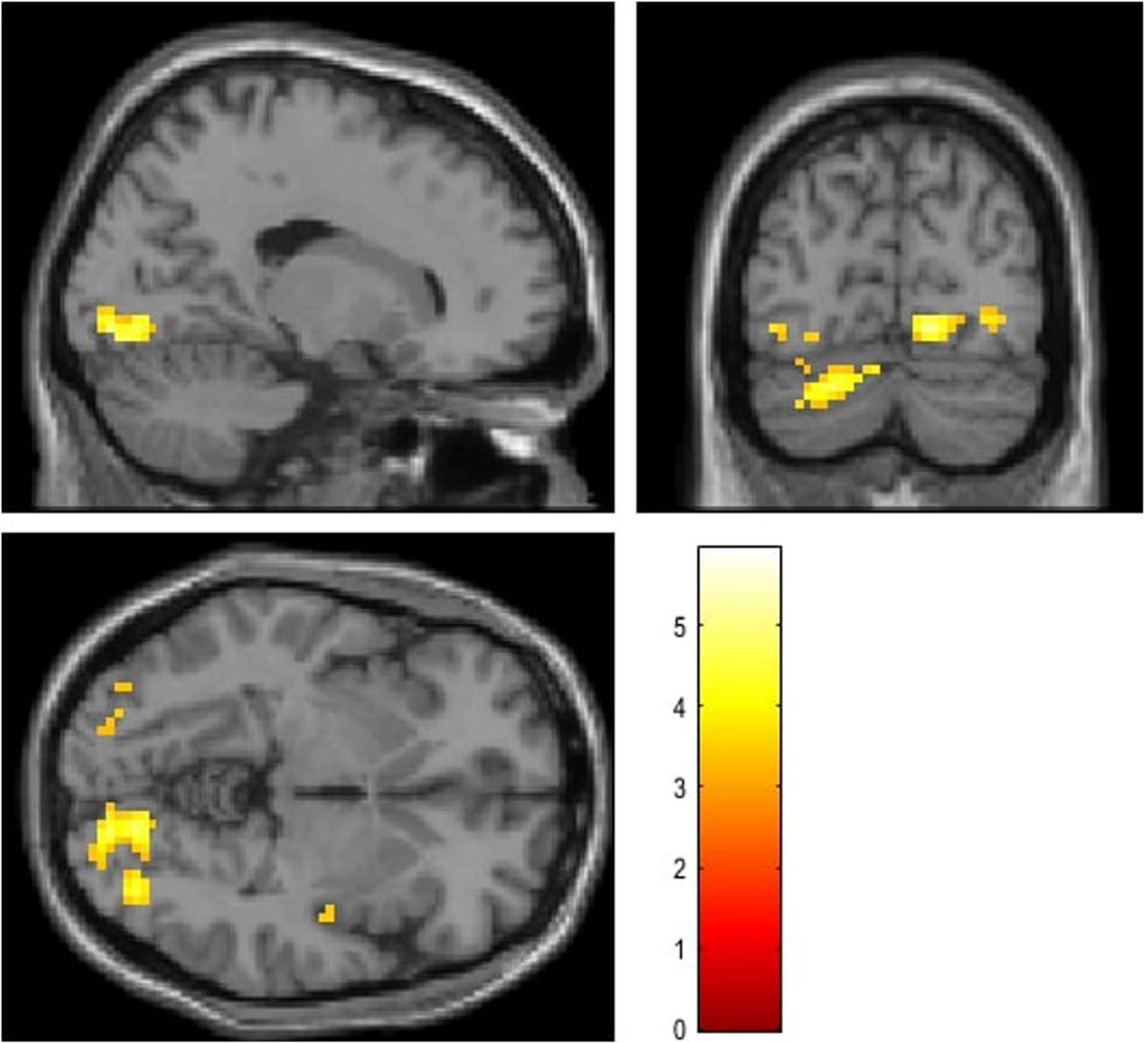
fMRI results of paired t-test for ES > MS for contrast correct rejection of lure > false alarms in the retrieval phase (*p* < .05, FDR corrected on the cluster level).

The impact of time-of-day (MS vs. ES) was found for the contrast correct rejection of lure > false alarms. In the retrieval phase the right lingual gyrus (MNI: 15 −79 −7; *t* = 5.94; k = 154 voxels) and left cerebellum (MNI: −24 −79 −28; *t* = 5.40; k = 145 voxels) were activated in the evening session compared to the morning one. The significance level was established at *p* < 0.05, FDR corrected on the cluster level (Fig. 4, Table 4).

**Table 4:** fMRI results of ES > MS for the contrast correct rejection of lure > false alarms in the retrieval phase (p < 0.05, FDR corrected on the cluster level).

Structure	MNI	k	T	p
Right lingual gyrus	15, −79, −7	154	5.94	0.033
Left cerebellum	−24, −79, −28	145	5.40	0.033

## Discussion

The present study demonstrates the dynamics of working memory processes—differential engagement of brain networks during veridical recognition and false memories—in both encoding and retrieval phases. Also, modulatory effects of time-of-day on neural activity were observed during the retrieval phase of a visually presented phonological short-term memory task. We employed an exploratory approach—Independent Component Analysis—to investigate phonological DRM illusion formation and true recognition. This approach allowed us to assess the dynamic involvement of brain networks in encoding and retrieval phases in relation to working memory performance.

Many experimental paradigms were utilized to investigate memory illusions (for the reviews, see: REF^34,38^). Numerous functional neuroimaging experiments that examine false recognition concentrate only on the retrieval phase as in the museum paradigm^66,67^, but some are focusing also on the encoding or/and storage processes (e.g. delayed-match-to-sample task). One of the paradigms, which allows investigating both the encoding and retrieval phases of the memorizing process, is utilized in our study the DRM paradigm.

Regarding the behavioral results, the differences in reaction times of the particular response types (the longest RTs for false alarms and correct rejection of lure probes, the shortest for correct rejection of negative probes) and between erroneous responses (misses, false alarms for lure and false alarms for negative probes) indicate the differences in information processing related to conflict monitoring^68^ and when participants make errors^69^. Our results are in line with previous studies^24,70^.

In the context of neural activity, our findings point out that the networks activated in the encoding phase of the visual phonological task comprise executive, right fronto-parietal, visual and sensory-motor networks, whereas deactivated networks include the anterior default mode network, auditory and dorsal attention networks. The activation of executive and right fronto-parietal networks in the encoding phase is in line with results obtained from studies investigating the neural basis of visual short memory showing that the prefrontal and parietal association cortex are crucial structures that encode information^71,72^. Visual and sensory-motor network activated during encoding provides highly selective tuning of stimulus-specific features (for the review, see: Ref^71^). Also, the deactivations of the anterior DMN, auditory and dorsal attention networks are consistent with previous results^73^. There is evidence that the deactivation of DMN during the encoding phase even enhances subsequent retrieval of maintained information^74^. Additionally, the analysis revealed activation of the medial visual network and deactivation of the insular network for hits, deactivation of the language network and activation of the medial visual network—for correct rejection of lure, and deactivation of insular and language network—for false alarms. We noticed the activity of the medial visual network (i.e. fusiform gyrus, BA 37) in the encoding phase that preceded accurate performance including hits and correct rejection of lure probes, whereas the engagement of the medial visual network during the encoding phase that preceded false alarms was not observed. The medial visual network is considered crucial in high-level visual processing for functionally-specialized processes related to object recognition, face perception, and language processing. A number of studies have also revealed the critical role of the left fusiform gyrus in both reading (e.g.^75,76^) and learning to read^77,78^. Seeing words engages a highly specialized part of the middle fusiform gyrus, known as the visual word form area (VWFA). The VWFA is typically lateralized in the left hemisphere and activated by visually presented words and pseudowords but not by auditory ones^79^. This region is thought to be sensitive to orthographic information^80–82^. The study by Glezer and colleagues (2015)^83^ employed fMRI-RA showed the VWFA as exclusively sensitive to orthography and not modulated by phonological information. Moreover, the study using PET^66^ as well as fMRI^84^, revealed the role of bilateral VWFA in pseudowords processing, suggesting reduced lateralization in the pseudowords than in the words condition. Rauschecker (2012)^85^ showed that the spatial pattern of VWFA response varies with stimulus position and that both the left and right middle fusiform gyri are involved in word processing, which together constitute a unified system that interfaces vision and language. Garoff and colleagues (2005)^86^ found that the fusiform gyrus is activated during the encoding phase bilaterally, dependent on specific (related to processing of stimuli details) or non-specific (related to global processing of stimuli) recognition. Furthermore, the study by Ardila, Bernal, and Rosselli (2015)^87^ utilizing a meta-analytic connectivity model to investigate the connectivity of the fusiform gyrus demonstrated the multimodality of this area. Two interconnected networks were found to be involved in different cognitive functions of language and visual processing. The interaction of the fusiform gyri with different brain areas points to the involvement of these structures in verbal working memory. This connectivity model also indicated a strong connection of fusiform gyri with the inferior frontal gyrus, a part of the language network.

At the retrieval phase, for all conditions, activation of the executive and right fronto-parietal networks and deactivation of the visual networks (medial and lateral) was observed. Additionally, for hits the activation of the left fronto-parietal networks was evident. Even though the left fronto-parietal network is involved in word processing, there are neuroimaging studies demonstrating the engagement of right frontal gyri and right parietal gyrus during the pseudowords reading^88,89^, as well as studies showing the involvement of right hemisphere in pseudowords visual processing^90,91^. The executive network comprising mainly of the prefrontal cortex and anterior cingulate cortex is responsible for cognitive control and optimizing goal-directed behaviors^92^. To sum up, the activation of executive and both right and left fronto-parietal networks supports true recognition. One of the most popular hypotheses in the functional neuroimaging experiments examining retrieval mechanisms of false memory is the sensory reactivation hypothesis, according to which the retrieval phase involves the reinstatement of processes that appeared in the encoding stage. This hypothesis assumes that true memory formation entails the activation of sensory areas (for the review, see: Ref^38^). In experiments using visual stimuli the increased activity of early visual regions was linked with true but not false memories formation, whereas late visual regions (including BA 37, i.e. fusiform gyrus) showed both activities linked with true and false memories^93^. Also, Slotnick and Schacter (2004)^94^ indicated that whereas early visual regions are primarily linked with true recognition (and seem to reflect sensory reactivation of previously seen stimuli), late visual structures were associated with both true and false recognition. The results of our short-term false memory study indicating deactivation of the visual networks (medial and lateral) at the retrieval phase for all conditions do not align with the sensory reactivation hypothesis. However, prior evidence supporting the hypothesis was obtained using long-term memory tasks and employing mainly visuo-spatial stimuli such as objects, photographs, or abstract shapes. In our short-term memory study even though the stimuli were presented visually, they were characterized by phonological similarity. Likewise, the results of an experiment employing stimuli that required mental imagery or orthographic-to-phonological transformation reported by Kahn, Davachi, and Wagner (2004)^95^ also do not support the sensory reactivation hypothesis. Recent evidence transforming the concept of sensory recruitment in short-term/working memory. As reviewed by Favila, Lee, and Kuhl (2020)^39^, during memory retrieval the transformation of content representations from visual regions to frontoparietal regions is revealed.

During correct rejection of lures—the deactivation of anterior and posterior default mode networks and activation of language network was observed. The deactivation of DMN during external task performance is well-established^96^, along with greater deactivations as cognitive load increases^97,98^. Likewise, our results demonstrated that the correct rejection of lures condition seems more demanding than the other conditions.

The activation of the language network (i.e. inferior frontal gyrus and superior temporal gyrus) in the retrieval phase, in the case of more demanding condition (correct rejection of lures) compared to the true recognition needs to be discussed. This network in the encoding phase was deactivated preceding the correct rejection of lures and false alarms. The inferior frontal gyrus is recognized as a structure involved in coding articulatory phonological features^99^. A meta-analysis based on neuroimaging studies on language processing showed the involvement of the inferior frontal gyrus not only for semantic and phonological processing but also for working memory (for the review, see: Ref^100^). As reviewed by Yi, Leonard, and Chang (2019)^101^, the superior temporal gyrus is considered one of the critical centers for phonological processing whereby meaningful linguistic features such as phonemes or syllables are extracted from the speech input. In light of our results, the dynamic interplay between medial visual and language networks for the more demanding probe (lure probe) supports the correct rejection of lures or false memory. Regarding the less demanding condition (encoding preceding hits), the activation of the executive and fronto-parietal network appears to be sufficient. Our results are in line with the dynamic-processing model of working memory, implicating the conversion and development of memory representations over time and showing that cognitive control induces engagement of different network to support goal-directed behavior^29^.

During the last decades, the false memory phenomenon has been extensively studied both in long-term and short-term/working memory, but the neural basis of false memory formation is rather underexplored. The main structures showing false recognition-related changes in the activity were the anterior cingulate cortex and the medial temporal gyrus (for a meta-analysis, see: Ref^34^), as well as fronto-parietal regions, superior temporal gyrus, and insula (for a meta-analysis, see: Ref^35^). Also, the amygdala activity was observed to differentiate false from true memories both at short and long time delays^102,103^, the same as the orbital cortices in long-term memory^102^. Yet, it should be noted that in these experiments, unlike in the current one, the morphed human faces were used, potentially increasing the stimuli saliency. Importantly, independently of the material type used in the experiment, the false memories were found to emerge based on both semantic and perceptual relatedness of these stimuli^104,105^. For instance, both semantically- and perceptually-related words were used^104,106^, the same as conceptually- and perceptually-related objects and scenes^93,105,107–109^. The concept of the dynamic-processing model of working memory utilized in our study allowed for a deeper insight into the neural basis of false memory formation.

Our findings highlight the complex interactions of memory representations during the encoding and retrieval phases, resulting in false memory, true recognitions, and correct rejection of lures. We demonstrated that phonological coding in the language network plays a role in working memory performance. Our results also have some parallels with the study by Zhu and colleagues employing DRM tasks in documenting complex interactions of memory representations during encoding and retrieval phases related to sensory modality^110^.

In the context of diurnal differences indicating an effect of circadian and/or homeostatic processes^111^ on veridical memory and memory distortions, this effect was not found. We observed the deactivations of the anterior and posterior DMN and the medial visual network with higher deactivation in the morning hours during the correct rejection of lure probes (at the retrieval phase). Previous studies have shown involvement of the DMN during visual n-back task^112^ as well as semantic and non-semantic declarative memory tasks^113^. Although here was studied short-term memory, we reported converging results, and additionally revealed involvement of the DMN that was dependent on the time-of-day. Blautzik et al. (2013)^114^ demonstrated the rhythmic connectivity patterns of DMN across a day, other evidence is provided by the study using regional homogeneity in resting-state fMRI, revealed the diurnal variations in the DMN activity^115^. Here, we confirmed the engagement of DMN in a specific condition of short-term memory task—the correct rejection of lure during retrieval, as well as the change of deactivation of default mode network dependent on the time-of-day, which could be explained by the effect of homeostatic process after a day full of activities^111^.

The GLM analysis also revealed diurnal differences in some brain regions, in the contrast correct rejection of lures vs. false alarms namely, in the left cerebellum and right lingual gyrus. During the last few decades, many studies have confirmed that the cerebellum is involved not only in motor control but also in many cognitive functions, including learning^116,117^, as well as in the phonological short-term memory^118^. Although the right cerebellum was considered responsible for language processing including phonological word retrieval^119^, Murdoch and Whelan (2007)^120^ demonstrated that left cerebellar lesions disrupt language processing and proposed model of bilateral involvement of the cerebellum in high-level linguistic processing. On the other hand, the right lingual gyrus is not typically recruited for short-term memory tasks but its function has been established for letter processing and words/pseudowords reading (for a meta-analysis, see: Ref^84^). It was shown that a decrease of right lingual gyrus involvement in word processing was correlated with semantic deficits in patients with ALS when they used Japanese sign-words^121^. Our results showed increased activation of this structure in the evening, which might be related to the information processing strategy that engaged the right hemisphere at this time of day as suggested by researchers exploring the diurnal and/or circadian variability of cognitive processes (e.g.^122–124^).

Taken together, the present study using Independent Component Analysis (ICA), DRM paradigm, and time-of-day protocol shed new light on the neural mechanism of false memory formation, dynamic process of veridical memory, and the modulatory effect of time-of-day on the retrieval process.

### Limitations and future directions

One limitation of our study is the lack of objective control or measurement of the fatigue of participants, which would allow us to show even more impact of the homeostatic process. Another limitation is the use of FDR correction, which is less conservative than FEW (family-wise error), nevertheless, FDR correction is often applied in neuroimaging research. Also, future studies could include analysis involving false alarms to unrelated probes, in particular a comparison of false alarms to related versus unrelated probes. This challenging comparison was not possible in our experiment due to very low error rate on unrelated, clearly distinct probes. When applied in future studies, it could provide further insight into the false memory phenomenon. Finally, future studies could use meaningful verbal material to explore if the patterns of network activation/deactivation are similar. Last but not least, the recent study by Spets and colleagues (2021)^125^ suggests sex differences in the neural correlates of retrieval processes, which is a relevant direction for future studies.

## Conclusions

To our knowledge, this is the first study on veridical and false memory using the ICA approach, which offers new insight into the neural mechanisms of false memory formation in short-term memory. The main findings illustrate a deficiency of medial visual network activity during the encoding phase, contributing to false memory formation in short-term memory. Our results related to true recognition and correct rejection of lures support the dynamic-processing model of working memory showing that cognitive control induces different dynamics of networks to support goal-directed behavior. The results related to the modulatory effect of time-of-day suggest another important, yet under-appreciated neurobiological factor that can influence the dynamics of working memory.

## Supplementary Information

Supplementary Information.

## Data Availability

Data can be made available from the corresponding author on reasonable request. Data were analyzed with open source toolboxes: SPM12 (Welcome Department of Imaging Neuroscience, UCL, London, UK), DPABI (V4.2) implemented on MATLAB (Mathworks, Inc., MA, USA) and GIFT software package (v4.0b; Medical Image Analysis Lab, University of New Mexico).

## Abbreviations

AM: Amplitude scale from Chronotype Questionnaire
BOLD: Blood oxygenation level dependent contrast
DMN: Default mode network
DRM: Deese–Roediger–McDermott
EHI: Edinburgh Handedness Inventory
EPI: Echo-planar imaging
ES: Evening session
ESS: Epworth Sleepiness Scale
ET: Evening type
FA: Flip angle
FDR: False discovery rate
fMRI: Functional magnetic resonance imaging
FOV: Field of view
GLM: General linear model
ICA: Independent component analysis
ICN: Intrinsic connectivity network
lfMRI: Laminar functional magnetic resonance imaging
MDL: Minimum description length
ME: Morningness/eveningness scale from Chronotype Questionnaire
MNI: Montreal Neurological Institute
MR: Magnetic resonance
MS: Morning session
MT: Morning type
PCA: Principal component analysis
PER3: Period circadian regulator 3
PSQI: Pittsburgh Sleep Quality Index
RT: Reaction time
ST-DRM: Short-term memory DRM
TE: Echo time
TR: Repetition time
VWFA: Visual word form area
