# Supplementary material for: Dynamics of working memory process revealed by independent component analysis in an fMRI study
Source: Sci Rep. 2023 Feb 18;13:2900. doi: 10.1038/s41598-023-29869-2 (PMC9938907; doi:10.1038/s41598-023-29869-2)
Supplement: Supplementary file 1 — Supplementary Information. [file 41598_2023_29869_MOESM1_ESM.pdf]

**Dynamics of working memory process revealed by independent component analysis in an fMRI study**

# SUPPLEMENTARY INFORMATION

Magdalena Fafrowicz[1,6], Anna Ceglarek[1*], Justyna Olszewska[2], Anna Sobczak[1], Bartosz Bohaterewicz[3], Monika Ostrogorska[4], Patricia Reuter-Lorenz[5], Koryna Lewandowska[1], Barbara Sikora-Wachowicz[1], Halszka Oginska[1], Magdalena Hubalewska-Mazgaj[7], Tadeusz Marek[1]

[1] Department of Cognitive Neuroscience and Neuroergonomics, Institute of Applied Psychology, Jagiellonian University, Krakow, Poland

[2] Department of Psychology, University of Wisconsin—Oshkosh, Oshkosh, WI, United States

[3] Department of Psychology of Individual Differences, Psychological Diagnosis and Psychometrics, Faculty of Psychology, SWPS University of Social Sciences and Humanities, Warsaw, Poland

[4] Chair of Radiology, Medical College, Jagiellonian University, Krakow, Poland

[5] Department of Psychology, University of Michigan, Ann Arbor, MI, United States

[6] Malopolska Centre of Biotechnology, Jagiellonian University, Krakow, Poland

[7] Department of Drug Addiction Pharmacology, Maj Institute of Pharmacology, Polish Academy of Sciences, Krakow, Poland

*corresponding author: Institute of Applied Psychology, Lojasiewicza Street 4, 30-348 Krakow, Poland, e-mail: aniaewacc@gmail.com

## 1. Chronotypes

Chronotype categorizes adults according to their morningness and eveningness (the propensity to be an early "lark" or a late "owl"), and displays itself as a tendency to sleep at a particular time during a 24-hour period [1,2,3]. In this paradigm, the experimental tasks are performed at optimal or nonoptimal time of day, depending on the chronotype (morning or evening). Circadian rhythms as well as homeostatic regulatory processes are responsible for endogenous control of human activity patterns and are associated with variations in biological parameters and cognitive performance [4,5]. Evidence has shown that evening-oriented people have shifted phases of physiological circadian rhythms toward later hours as compared to the morning-oriented ones [6,7]. Biological parameters not only include the sleep-wake cycle but also body temperature as well as the production of melatonin and cortisol, while cognitive performance effects pertain to attention, cognitive speed and memory. Previous studies confirmed better performance in the morning hours of morning types and the opposite tendency for evening types, so called synchrony effect [4,8,9].

## Results related to chronotypes

We found no impact of chronotype on the networks' organization during task performance nor on the differences between activity of brain regions as well as on the reaction times. Only one behavioral results measure (sensitivity) revealed an effect of chronotypes, the evening types showing greater reliance on phonological gist, which is defined as thematic relations between phonologically similar items [10]. Originally, gist-based memory was introduced by Brainerd and Reyna (1998) [11] and is defined as understanding a bottom line meaning, or in other words, possessing fuzzy representations of a past event. In the context of our stimuli, it is probable that participants while encoding pseudowords tended to label them and eventually found a word from their lexicon that was the closest in its sound to the studied pseudoword [12]. At the time of retrieval they might have based their decisions not only on the phonological similarities but also on self-generated meaning. It seems that the evening types rely more on global rather than a detailed analysis of the stimulus during the decision-making process [13].

References

[1] Goldstein, D., Hahn, C. S., Hasher, L., Wiprzycka, U. J. & Zelazo, P. D. Time of day, intellectual performance, and behavioral problems in Morning versus Evening type adolescents: Is there a synchrony effect? *Pers. Individ. Differ*. **42(3)**, 431–440. https://doi.org/doi:10.1016/j.paid.2006.07.008 (2007).

[2] Goel, N., Basner, M., Rao, H. & Dinges, D. F. Circadian rhythms, sleep deprivation, and human performance. *Prog Mol Biol Transl Sci*. **119**, 155-190 (2013).

[3] Fafrowicz, M. *et al*. Beyond the low frequency fluctuations: morning and evening differences in human brain. *Front. Hum. Neurosci*. **13**, 288. https://doi.org/10.3389/fnhum.2019.00288 (2019).

[4] Schmidt, C., Collette, F., Cajochen, C. & Peigneux, P. A time to think: circadian rhythms in human cognition. *Cogn. Neuropsychol*. **24**, 755–789. https://doi.org/10.1080/02643290701754158 (2007).

[5] Hardiess, G., Meilinger, T. & Mallot, H. A. The International Encyclopedia of the Social and Behavioral Sciences (ed. James Wright) (Elsevier, 2015).

[6] Gibertini, M., Graham, C. & Cook, M. R. Self-report of circadian type reflects the foxphase of the melatonin rhythm. *Biol. Psychol*. **50**, 19–33. https://doi.org/10.1016/s0301-0511(98)00049-0 (1999).

[7] Bailey, S. L. & Heitkemper, M. M. Circadian rhythmicity of cortisol and body temperature. Morningness-eveningness effects. *Chronobiol. Int*. **18**, 249–261. https://doi.org/10.1081/cbi-100103189 (2001).

[8] Schmidt, C. *et al*. Pushing the Limits: Chronotype and Time of Day Modulate Working Memory-Dependent Cerebral Activity. *Front. Neurol*. **6**. https://doi.org/10.3389/fneur.2015.00199 (2015).

[9] Valdez, P., Ramírez, C. & García, A. Circadian rhythms in cognitive performance: implications for neuropsychological assessment. *Chronophysiol. Ther*. **2**, 81–92. https://doi.org/10.2147/cpt.s32586 (2012).

[10] Holliday, R. E. & Weekes, B. S. Dissociated developmental trajectories for semantic and phonological false memories. *Memory* **14(5)**, 624–636. https://doi.org/10.1080/09658210600736525 (2006).

[11] Brainerd, C. J., & Reyna, V. F. Fuzzy-Trace Theory and Children's False Memories. *J. Exp. Child Psychol*. **71(2)**, 81-129. (1998).

[12] Schweickert, R. A multinomial processing tree model for degradation and redintegration in immediate recall. *Mem Cognit*. **21**, 168-175. (1993).

[13] Reyna, V. F. A new intuitionism: Meaning, memory, and development in Fuzzy-Trace Theory. *Judgm*. **7(3)**, 332–359. (2012).

2. Genotyping procedure

The DNA was isolated from buccal swabs using DNA GeneMATRIX Swab-Extract DNA Purification Kit (EURx, Gdańsk, Poland) following the manufacturer's protocol. VNTR polymorphism in PER3 gene was analyzed with PCR. 2 µl of each DNA sample was amplified in the final reaction volume of 20 µl containing: 2 µl of 10x Pfu buffer (EURx, Gdańsk, Poland), 0.5 U of Pfu polymerase (EURx, Gdańsk, Poland), 0.5 µM of forward/reverse primer and 0,2 mM dNTP mix. Following amplification, each PCR reaction product was analyzed by electrophoresis in agarose gel (1.8% agarose, 0.5x TBE) stained with ethidium bromide and documented in a gel documentation system.

Table S1. The selected intrinsic connectivity networks with structures that belong to certain networks and MNI coordinates of the maximum peak of the signal. LH - left hemisphere; RH - right hemisphere.

| | network | structure | MNI coordinates of the peak |
|---|---|---|---|
| 1. | anterior DMN | medial prefrontal cortex | 8,59,19 (RH) |
| | | anterior cingulate gyrus | 5,38,18 (RH) |
| 2. | posterior DMN | angular gyrus | 43,-61,39 (RH) |
| | | posterior cingulate gyrus | -5,-44,29 (LH) |
| | | precuneus | 9,-57,42 (RH) |
| 3. | left fronto-parietal network | middle frontal gyrus | -38,41,24 (LH) |
| | | inferior parietal lobule | -49,-43,47 (LH) |
| 4. | right fronto-parietal network | middle frontal gyrus | 36,23,44 (RH) |
| | | inferior parietal lobule | 50,-42,52 (RH) |
| 5. | auditory network | superior temporal gyrus | -45,-7,-10 (LH) |
| | | Heschl's gyrus | -40,-22,8 (LH) |
| 6. | language network | inferior frontal gyrus | -40,24,17 (LH) |
| | | superior temporal gyrus | -56,-37,17 (LH) |
| 7. | medial visual network | fusiform gyrus | 30,-60,-11 (RH) |
| 8. | lateral visual network | superior occipital gyrus | 19,-92,24 (RH) |
| | | middle occipital gyrus | -32,-81,21 (LH) |
| 9. | occipital pole | inferior occipital gyrus | 39,-84,-6 (RH) |
| 10. | insular network | insula | 43,12,-5 (RH) |
| 11. | sensory-motor network | precentral gyrus | -33,-18,64 (LH) |
| | | supplementary motor area | -9,4,62 (LH) |
| 12. | executive network | middle frontal gyrus | 35,24,45 (RH) |
| | | anterior cingulate gyrus | 9,27,24 (RH) |
| 13. | dorsal attetntion network | intraparietal sulcus | -23,-70,46 (LH) |
